# Effect of bladder volume on measured intravesical pressure: a prospective cohort study

**DOI:** 10.1186/cc4962

**Published:** 2006-07-06

**Authors:** Manu LNG Malbrain, Dries H Deeren

**Affiliations:** 1 Intensive Care Unit, Ziekenhuisnetwerk Antwerpen Campus Stuivenberg, Lange Beeldekensstraat, B-2060 Antwerp, Belgium; 2 Department of Internal Medicine and Intensive Care Medicine, Ziekenhuisnetwerk Antwerpen Campus Stuivenberg, Lange Beeldekensstraat, B-2060 Antwerp, Belgium

## Abstract

**Introduction:**

Correct bedside measurement of intra-abdominal pressure (IAP) is important. The bladder method is considered as the gold standard for indirect IAP measurement, but the instillation volumes reported in the literature vary substantially. The aim of this study was to evaluate the effect of instillation volume on intra-bladder pressure (IBP) as an estimation for IAP in critically ill patients.

**Methods:**

In this prospective cohort study in 13 sedated and mechanically ventilated patients, we used a revised closed system repeated measurement technique for measurement of IBP. After the system was flushed, IBP was measured with 25 ml increments up to 300 ml. The absolute bias for each volume was calculated as IBP at a given volume minus IBP at zero volume.

**Results:**

In total, 30 measurement sets were performed (mean 2.3 per patient). The median IBP at 25 ml was already significantly higher than IBP at zero volume (7.5 versus 6 mmHg). There was no correlation between IBP at zero volume and absolute IBP bias at any bladder volume. Median absolute IBP bias was 1.5 mmHg at 50 ml; 2.5 mmHg at 100 ml; 5.5 mmHg at 150 ml; and up to 11 mmHg at 300 ml.

**Conclusion:**

Larger instillation volumes than the usually recommended 50 ml to estimate IAP by bladder pressure may cause clinically relevant overestimation of IAP. Small volumes to a maximum of 25 ml, enough to create a fluid column and to remove air, may be sufficient.

## Introduction

The occurrence of intra-abdominal hypertension (IAH) during intensive care unit (ICU) stay is an independent predictor for mortality [1-3] and IAH may cause injury to various organs [4]. Recently, we found that increases in intra-abdominal pressure (IAP), even at low levels, are associated with increases in intracranial pressure and decreases in cerebral perfusion pressure in patients with non-traumatic brain injury [5]. Correct bedside measurement of IAP is important because it allows the ICU physician to evaluate the effect of surgical or non-surgical decompression on IAP. Furthermore, IAP is a physiological parameter that may guide interventions to improve the haemodynamic or respiratory status [4,6].

The World Society on Abdominal Compartment Syndrome (WSACS) [7] stated that until other methods are validated, the bladder method is considered as the gold standard for indirect IAP measurement (the gold standard for direct measurement being intraperitoneal pressure measurement) [4,7]. However, the instillation volumes reported in the literature for bladder pressure measurement are not uniform and range from 50 up to 300 ml [8,9]. Instillation of large volumes may raise the intrinsic bladder pressure and cause overestimation of IAP.

The aim of this study was to evaluate the effect of instillation volume on intra-bladder pressure (IBP) as an estimation for IAP in critically ill patients. Parts of this study were presented at the 16th Annual congress of the European Society of Intensive Care Medicine in Amsterdam, The Netherlands (6 to 8 October 2003) [10].

## Materials and methods

### Study design, setting and participants

We performed a prospective cohort study at the 12 bed medical ICU of the Stuivenberg hospital. The study was conducted in accordance with the study protocol, the Declaration of Helsinki and applicable regulatory requirements. The local Institutional Review Board and Ethics Committee approved the protocol. In view of the nature of the study, which did not demand a deviation from standard clinical ICU care, informed consent from the patient or the next of kin was not essential. All sedated and ventilated patients in whom bladder pressure was measured to estimate IAP were eligible for the study. Patients were excluded if they had bladder injury or if they were haemodynamically or respiratorally unstable. IAH and abdominal compartment syndrome (ACS) were defined according to the WSACS suggested consensus definitions on their website [7].

### Study protocol and materials

We used the revised closed system repeated measurement technique for measurement of bladder pressure [8]. In this technique, a ramp with three stopcocks is inserted in the drainage tubing connected to a Foley catheter. A standard infusion set is connected to a bag of 1,000 ml of saline and attached to the first stopcock. A 60 ml syringe is connected to the second stopcock and the third stopcock is connected to a pressure transducer via rigid tubing. The system is flushed with saline to remove air, and the pressure transducer is zeroed at the symphysis pubis. To measure IBP, the bladder is completely emptied and the urinary drainage tubing is clamped distal to the ramp-device. The desired amount of saline is aspirated from the bag into the syringe and then instilled in the bladder. After opening the stopcocks to the pressure transducer, the IBP can be read from the bedside monitor. To confirm correct measurement, a rapid flush test, inspection of respiratory pressure variations and an oscillation test were performed before every measurement.

After the system was flushed, baseline IBP was measured without instilling extra volume. Then, IBP measurements were performed with 25 ml increments up to 300 ml and with 25 ml decrements until baseline. Each instillation was followed by a one minute equilibration period. The IBP was read as an integer value recorded at end-expiration. All IBP values were transduced to the same mark and type of bedside monitor (Spacelabs, Issaquah, Washington, USA). Each patient underwent repeated measurement series. All measurements were performed by the same observer to limit interobserver variability. Mean values were used for statistical analysis.

### Analysis

For each bladder volume in each patient, mean IBP was recorded and absolute bias was calculated. The absolute bias for each volume was calculated as IBP at a given volume minus IBP at zero volume. Although only the absolute bias is clinically relevant in IAP estimation, the relative bladder pressure bias was also calculated. The relative bias for each volume was calculated as the IBP at a given volume minus IBP at zero volume divided by IBP at zero volume and expressed as a percentage. We used the Shapiro-Wilk *W *test to test for normality, weighted correlation (because of repeated measurements) to test for correlation between IBP at zero volume and absolute bladder pressure bias, and the Wilcoxon matched pairs signed rank sum test to test for differences between IBP at different bladder volumes. Pearson correlation coefficient and Bland and Altman analysis was used to compare IBP at zero and 50 ml of priming volume.

## Results

### Patients

We studied 13 intubated and mechanically ventilated patients (eight women, five men). Mean age was 69.2 ± 13.5 years, mean body mass index was 24.5 ± 3.9, mean simplified acute physiology (SAPS II) score was 60.4 ± 13.2, mean sequential organ failure assessment (SOFA) score was 9.6 ± 3.5 and mean acute physiology and chronic health evaluation (APACHE-II) score was 27.8 ± 9.8. In total, 30 measurement series were performed. The number of measurements in each patient was 2.3 ± 0.5 (range 2 to 3).

### Bladder pressures

**Figure 1:**
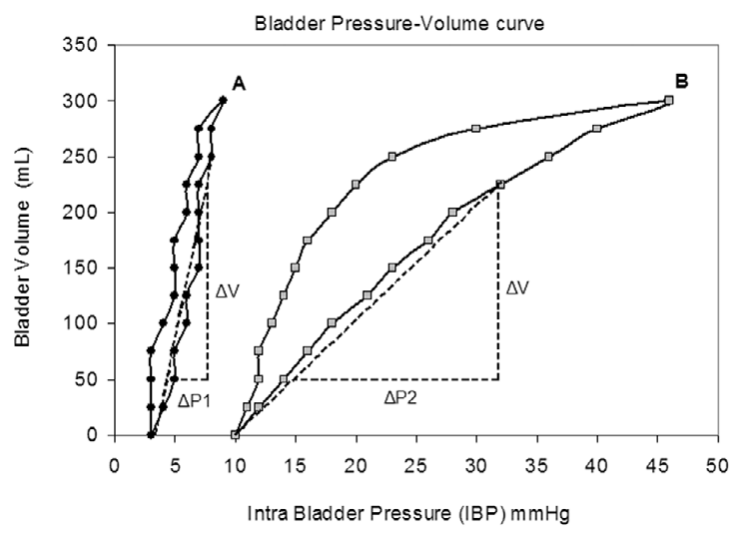
Example of two bladder pressure volume (PV) curves. Curve A (black circles) shows the bladder PV curve in a patient with a normal bladder compliance: baseline intra-bladder pressure (IBP; at empty bladder) was 3 mmHg; bladder compliance was 38.3 ml/cm H_2_O (ΔV/ΔP1). Curve B (grey squares) shows the bladder PV curve in another patient with a low bladder compliance: baseline IBP (at empty bladder) was 10 mmHg; bladder compliance was 10.2 ml/cm H_2_O (ΔV/ΔP2). The PV curves are shown with the inflation/filling limb (right hand side) and the deflation/emptying limb (left hand side).

**Figure 2:**
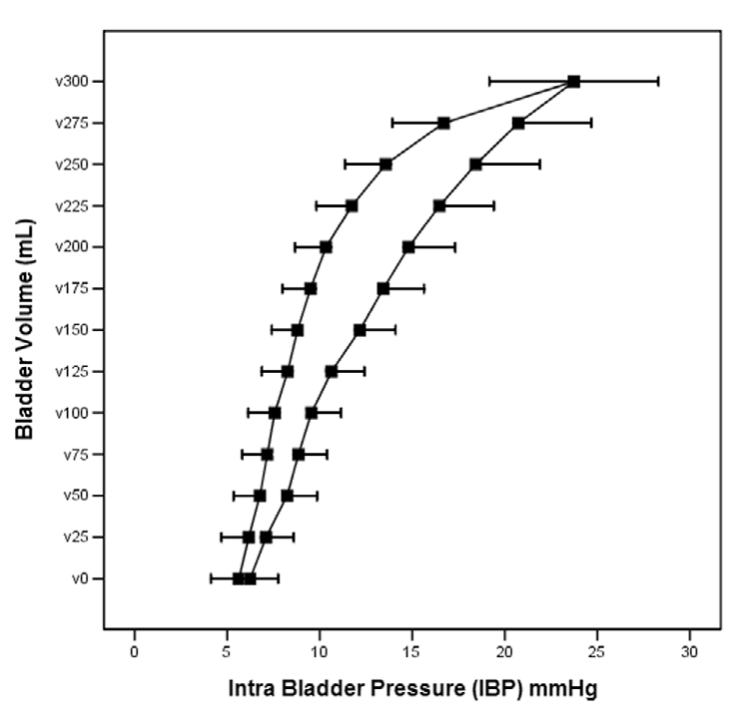
Curve fit of the mean intra-bladder pressures (IBPs) on the X-axis versus the instilled bladder volume (Y-axis) from all patients. The 'inflation' (right-hand side) and 'deflation' (left hand side) pressure volume (PV) curve is plotted as a curve fit of the means of the 30 measurements. Hysteresis can be seen, as with respiratory PV curves. No clear lower inflection point can be identified; via extrapolation, an upper inflection point was found at a bladder volume of 150 ml to 175 ml on the inflation limb. Error bars show 95% confidence intervals.

Mean bladder pressures at different bladder instillation volumes are summarized in Table 1. The IBP at 25 ml was already significantly higher than IBP at zero volume (*p *= 0.007 for 25 ml; *p *= 0.001 for 50 ml; and *p *< 0.0001 for all other comparisons). Hence, in each patient, bladder pressure volume (PV) curves could be obtained, illustrating the relative bladder compliance at that moment (Figure 1). The PV curve fit of the mean showed a linear increase in IBP with ongoing filling, without a clear inflection point (Figure 2).

**Figure 3:**
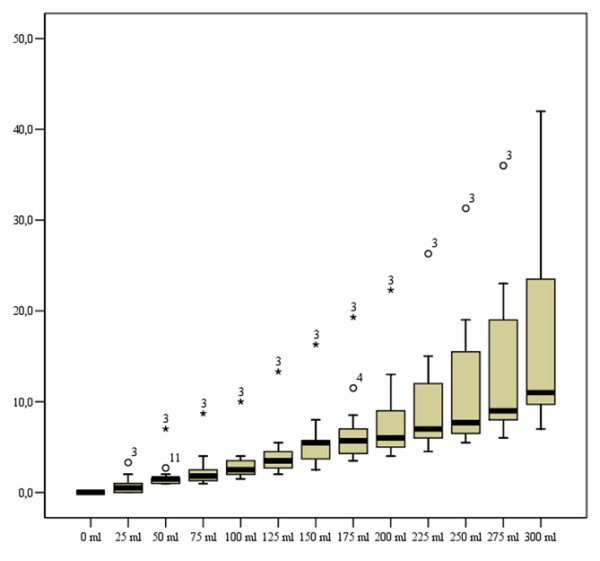
Boxplot of absolute bladder pressure bias (Y-axis, mmHg) against instilled bladder volume (X-axis). The absolute bias for each volume was calculated as intra-bladder pressure (IBP) at a given volume minus IBP at zero volume. Each box shows the median values and quartiles for absolute bladder pressure bias. Outliers (cases with values between 1.5 and three box lengths from the upper or lower edge of the box) are denoted with circles. Extreme cases (cases with values more than three box lengths from the upper or lower edge of the box) are denoted with asterisks. For both outliers and extreme cases, the patient number is shown above the symbol.

There was no correlation between IBP at zero volume (baseline) and absolute IBP bias at any bladder volume (Pearson's correlation coefficient -0.036 to -0.290; *p *> 0.05). These data are shown in the pressure-volume boxplots in Figure 3. The observed data of absolute bias for each bladder volume were not normally distributed (Shapiro-Wilk *W *statistic 0.550 to 0.849; *p *< 0.05). Therefore, the median values of absolute IBP bias are more representative than the mean values. Median absolute IBP bias was 1.5 mmHg at 50 ml; 2.5 mmHg at 100 ml; 5.5 mmHg at 150 ml; and up to 11 mmHg at 300 ml.

**Figure 4:**
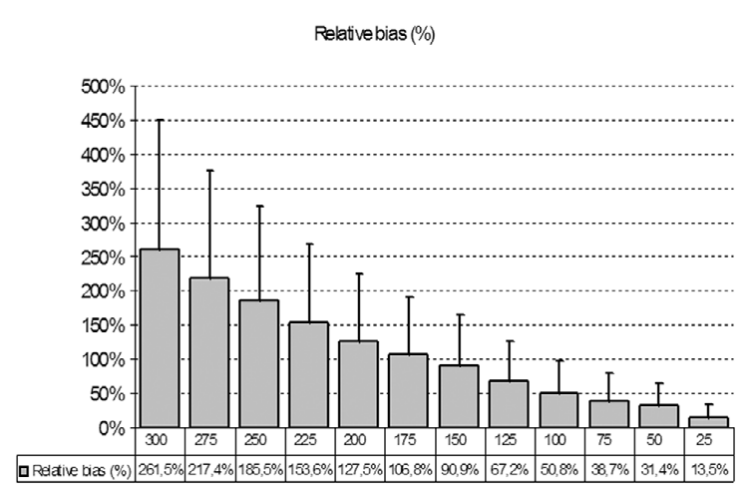
Bar graph of relative bladder pressure bias (Y-axis, %) against instilled bladder volume (X-axis, ml). The relative bias for each volume was calculated as the intra-bladder pressure (IBP) at a given volume minus IBP at zero volume divided by IBP at zero volume and expressed as a percentage. Each bar shows the mean value with error bar (standard deviation) for relative bladder pressure bias at different instillation volumes.

The relative bladder pressure bias could only be calculated for 11 patients (since the IBP at zero volume was close to zero mmHg in 2 patients, leading to an excessively high relative bias). Figure 4 shows the relative bias related to the different instillation volumes. There was no correlation between baseline IBP and relative bladder pressure bias at any volume.

Pearson correlation between IBP obtained at zero and 50 ml of priming fluid was good with an R2 of 0.8; however, the analysis according to Bland and Altman showed a mean bias of 1.5 ± 1.8 mmHg, with large limits of agreement (from -2.1 to 5.1 mmHg).

## Discussion

The main result of our study is that large instillation volumes are associated with higher IBP. This may cause overestimation of IAP. The increase in IBP was already statistically significant at the smallest instillation volume (25 ml), but only became clinically relevant at a volume of 75 ml for most of the patients. For one patient, however, the PV curve was flattened and shifted to the right, with overestimation being obvious even at the smallest instillation volumes (Figure 1).

There are two explanations for the rise in IBP after instillation of large volumes. First, the bladder wall is not merely a membrane that transfers pressure from the intra-abdominal space to the bladder content. Bladder wall compliance differs between patients and depends on several factors like age, BMI, filling status and bladder perfusion/ischemia [11]. Second, IAP reflects the relationship between the intra-abdominal volume and abdominal compliance. In the physiological range, small volume increases do not cause substantial IAP increases until a point of decompensation, after which each small increase in volume results in a relatively large increase in IAP. If injury causes an enlargement of one of the intra-abdominal contents, abdominal compliance can be reduced. In that situation, it is possible that instillation of a large amount of fluid in the bladder leads to an iatrogenic IAP increase. We did not, however, find a correlation between IBP at zero volume and absolute or relative IBP bias.

Our results agree with the data of Fusco and colleagues [12] who compared direct IAP measurements during laparoscopy with IBPs. IBPs at larger instillation volumes were systematically higher than at smaller volumes, and 'average bias' was lowest at the minimal instillation volume ('average bias' = directly measured IAP minus IBP = -0.79 mmHg). Instillation of 50 ml saline resulted in an 'average bias' of -3.7 mmHg. However, calculation of correlation between direct IAP and IBP was jeopardized by two factors. First, IAP was adjusted to a specific value by abdominal insufflation after, but not before, each bladder instillation. This means that iatrogenic IAP increases by bladder instillation were masked. Second, correlation was calculated with Pearson correlation coefficients for the entire 222 data set without accounting for the fact that there were only 37 patients, indicating that on average 6 repeated measurements were performed in each patient.

The clinical implications of our results are that instillation of a large volume for IAP estimation may lead to IAP overestimation and misdiagnosis of IAH or ACS (Table 2). This may lead to unnecessary and even harmful interventions, underdiagnosis of other pathology and prolonged ICU stay. In the absence of strong data that demonstrate a better correlation of directly measured IAP with IBP at one specific volume, we suggest to measure IBP after priming the system with a small amount of saline (maximum 25 ml), enough to create a fluid column and to remove air. Our results are in accordance with those observed by De Waele and colleagues [13], who also suggested recently that priming the bladder with 10 ml of saline may be sufficient for estimation of IAP. In their paper, the percentage difference (or relative bias) for IAP50 (IAP obtained after instaillation of 50 ml) and IAP100 (IAP obtained after installation of 100 ml for each patient was calculated as: (IAP50 or IAP100 - IAPminimum)/IAPminimum. They found that the mean percentage difference was 21% for IAP50 and 40% for IAP100. This is in accordance with our findings, with a relative bias defined as (IAP50 or IAP100 - IAP0)/IAP0 of 31.4% and 50.8% at instillation volumes of 50 and 100 ml, respectively (Figure 4). However, it is difficult to compare our data further since our mean IAP0 was only 6.6 mmHg compared to 12.8 mmHg in the study by De Waele and colleagues. Also, their incidence of IAH was 60%, 75% and 85% at instillation volumes of 10, 50 and 100 ml, respectively, versus 7.1%, 23.1% and 23.1% in our study. Also in the study by De Waele and colleagues, only one set of measurements was performed per patient.

Our study has limitations. The number of patients studied was relatively small. We only compared IBPs with each other and not with directly measured IAP. The ideal study, that is, a comparison between direct measured IAP and IBP at different priming volumes, should include a Bland-Altman analysis as recommended by the WSACS. Furthermore, our results are only applicable to our observed values of IAP. Also as pointed out by De Waele and colleagues, the temperature, rate of infusion or volume of the instillate may also cause increased detrusor tone. This might be resolved by using aslow infusion of saline at body temperature. Another limitation may be that the bladder needs more time (longer than the one minute we used in this study) to adapt, and that an IAP recording should only be done minutes after instilling saline into the bladder.

## Conclusion

Large instillation volumes of 75 ml or more to estimate IAP by IBP may cause clinically relevant overestimation of IAP and hence a false increase in the prevalence and incidence of IAH and ACS. Bladder compliance varies within and between patients. Small volumes to a maximum of 25 ml, enough to create a fluid column and to remove air, should be used.

## Key messages

• 	Instilling over 50 ml of saline into the bladder may overestimate actual IAP

• 	Instilling over 50 ml may falsely increase the prevalence and incidence of IAH and ACS

• 	25 ml may be enough to prime the bladder for estimation of IAP

• 	Bladder compliance varies between and within ICU patients

• 	A uniform, standardised, accurate and reproducible IAP measurement method is needed for multiple centre study purposes

## Abbreviations

ACS = abdominal compartment syndrome; IAH = intra-abdominal hypertension; IAP = intra-abdominal pressure; IBP = intra-bladder pressure; ICU = intensive care unit; PV = pressure volume; WSACS = World Society on Abdominal Compartment Syndrome.

## Competing interests

MM is a member of the medical advisory board of Pulsion Medical Systems. MM is the founding president of the WSACS.

## Authors' contributions

MM conceived of the study and its design, participated in the data collection, helped to draft the manuscript and made the final review. DD participated in the data collection, performed the statistical analysis and drafted the manuscript.

## Figures and Tables

**Table 1:** Mean intra-bladder pressure, expressed in mmHg at different bladder instillation volumes, per individual patient obtained during bladder filling

Patient	Bladder Instillation Volume												
	0 ml	25 ml	50 ml	75 ml	100 ml	125 ml	150 ml	175 ml	200 ml	225 ml	250 ml	275 ml	300 ml

1	7	7.5	8.5	8.8	9.8	10	11	11.5	12	13	13.5	14	15.5
2	7	7.5	8	9	9	9.5	10	10.5	11	11.5	12.5	13	14
3	6	9.3	13	14.7	16	19.3	22.3	25.3	28.3	32.3	37.3	42	48
4	0.5	1.5	2.5	4.5	4.5	6	8.5	12	13.5	15	17	22	27
5	4	4	5.3	5.7	6	7.7	9.7	9.7	10	11	11.7	12.7	13.7
6	0	0.5	1.5	2.5	3	3.5	4	5	6	7	7	9	11
7	12.7	13.3	13.7	14	14.7	15.3	16.3	17	17.7	18.7	19.7	21	23.3
8	6	6	7	7	8	8	8.5	9.5	10	11	12	14	15
9	6	6.5	7.5	7.5	8.5	9.5	11.5	13	15	18	21.5	25	29.5
10	12	12	13	13	13.5	15	17.5	20.5	23.5	27	31	35	39.5
11	4	6	6.7	7	8	8.7	10.3	11	12.3	13.7	15.7	17.3	21.3
12	8.5	8.5	9.5	9.5	10	10.5	11.5	12	13	13.5	15	16.5	18.5
13	6.5	7.5	8	9	10	11	12	13	15.5	17.5	20.5	23	26.5
Mean	6.2	6.9	8	8.6	9.3	10.3	11.8	13.1	14.4	16.1	18	20.3	23.3
Median	6	7.5	8	8.8	9	9.5	11	12	13	13.7	15.7	17.3	21.3

**Table 2:** Incidence of intra-abdominal hypertension and abdominal compartment syndrome related to different instillation volumes for IAP estimation

	Bladder Instillation Volume												
	300 ml	275 ml	250 ml	225 ml	200 ml	175 ml	150 ml	125 ml	100 ml	75 ml	50 ml	25 ml	0 ml

IAH	92.3%	92.3%	84.6%	69.2%	69.2%	53.8%	30.8%	23.1%	23.1%	23.1%	23.1%	15.4%	15.4%
ACS	53.8%	46.2%	30.8%	15.4%	15.4%	15.4%	7.7%	0.0%	0.0%	0.0%	0.0%	0.0%	0.0%

The percentages were calculated using the WSACS suggested thresholds and definitions [7] and were based on the mean intra-abdominal pressure (IAP) values obtained in each patient.

## References

[B1] Malbrain ML, Chiumello D, Pelosi P, Bihari D, Innes R, Ranieri VM, Del Turco M, Wilmer A, Brienza N, Malcangi V (2005). Incidence and prognosis of intraabdominal hypertension in a mixed population of critically ill patients: a multiple-center epidemiological study. Crit Care Med.

[B2] Balogh Z, McKinley BA, Holcomb JB, Miller CC, Cocanour CS, Kozar RA, Valdivia A, Ware DN, Moore FA (2003). Both primary and secondary abdominal compartment syndrome can be predicted early and are harbingers of multiple organ failure. J Trauma.

[B3] Biancofiore G, Bindi ML, Romanelli AM, Boldrini A, Consani G, Bisa M, Filipponi F, Vagelli A, Mosca F (2003). Intra-abdominal pressure monitoring in liver transplant recipients: a prospective study. Intensive Care Med.

[B4] Malbrain ML, Deeren D, De Potter TJ (2005). Intra-abdominal hypertension in the critically ill: it is time to pay attention. Curr Opin Crit Care.

[B5] Deeren D, Dits H, Malbrain MLNG (2005). Correlation between intra-abdominal and intracranial pressure in nontraumatic brain injury. Intensive Care Med.

[B6] Gattinoni L, Chiumello D, Carlesso E, Valenza F (2004). Bench-to-bedside review: chest wall elastance in acute lung injury/acute respiratory distress syndrome patients. Crit Care.

[B7] WSACS EC World Society on Abdominal Compartment Syndrome. http://www.wsacs.org.

[B8] Malbrain ML (2004). Different techniques to measure intra-abdominal pressure (IAP): time for a critical re-appraisal. Intensive Care Med.

[B9] Malbrain M, Jones F, Ivatury R, Cheatham M, Malbrain M, Sugrue M (2006). Intra-abdominal pressure measurement techniques. Abdominal Compartment Syndrome.

[B10] Malbrain MLNG, Deeren D, Darquennes K, Van Mieghem N, Verbrugghe W, Daelemans R, Lins R (2003). Estimating optimal bladder volume for intra-abdominal pressure measurement by bladder PV-curves. Intensive Care Med.

[B11] Kershen RT, Azadzoi KM, Siroky MB (2002). Blood flow, pressure and compliance in the male human bladder. J Urol.

[B12] Fusco MA, Martin RS, Chang MC (2001). Estimation of intra-abdominal pressure by bladder pressure measurement: validity and methodology. J Trauma.

[B13] De Waele J, Pletinckx P, Blot S, Hoste E (2006). Saline volume in transvesical intra-abdominal pressure measurement: enough is enough. Intensive Care Med.

